# Use of a flexible optical fibre bundle to interrogate a Fabry–Perot sensor for photoacoustic imaging

**DOI:** 10.1364/OE.27.037886

**Published:** 2019-12-13

**Authors:** Rehman Ansari, Edward Z. Zhang, Adrien E. Desjardins, Anna L. David, Paul C. Beard

**Affiliations:** 1 Department of Medical Physics and Biomedical Engineering, University College London, Gower Street, WC1E 6BT, UK; 2 Wellcome/EPSRC Centre for Interventional and Surgical Sciences, University College London, 43-45 Foley Street, London W1W 7TS, UK; 3 Elizabeth Garrett Anderson Institute for Women’s Health, University College London, 74 Huntley Street, London WC1E 6AU, UK

## Abstract

Photoacoustic imaging systems based on a Fabry Perot (FP) ultrasound sensor that is read-out by scanning a free-space laser beam over its surface can provide high resolution photoacoustic images. However, this type of free-space scanning usually requires a bulky 2-axis galvanometer based scanner that is not conducive to the realization of a lightweight compact imaging head. It is also unsuitable for endoscopic applications that may require complex and flexible access. To address these limitations, the use of a flexible, coherent fibre bundle to interrogate the FP sensor has been investigated. A laboratory set-up comprising an x-y scanner, a commercially available, 1.35 mm diameter, 18,000 core flexible fibre bundle with a custom-designed telecentric optical relay at its distal end was used. Measurements of the optical and acoustic performance of the FP sensor were made and compared to that obtained using a conventional free-space FP based scanner. Spatial variations in acoustic sensitivity were greater and the SNR lower with the fibre bundle implementation but high quality photoacoustic images could still be obtained. 3D images of phantoms and *ex vivo* tissues with a spatial resolution and fidelity consistent with a free-space scanner were acquired. By demonstrating the feasibility of interrogating the FP sensor with a flexible fibre bundle, this study advances the realization of compact hand-held clinical scanners and flexible endoscopic devices based on the FP sensing concept.

## 1. Introduction

Photoacoustic tomography (PAT) is a non-invasive imaging technique that combines the high spatial resolution of ultrasonography with high optical absorption contrast to visualize the structure and function of tissues to centimeter scale depths [1–3]. PAT utilizes the specific absorption features of chromophores such as haemoglobin to visualise vascular structures [4], to obtain physiologically relevant functional information such as oxygen saturation [5,6], or to exploit lipid absorption to characterise vulnerable atherosclerotic plaques [7,8]. A variety of photoacoustic imaging scanners based on the use of piezoelectric detectors have been demonstrated [1]. However, for short-range high-resolution imaging in widefield PA tomography mode [1], the limited acoustic bandwidth and insufficiently fine spatial sampling of piezoelectric based detection schemes can compromise image quality. An alternative detection method that can address these limitations is based upon the use of a Fabry Perot (FP) polymer film ultrasound sensor [9]. In this approach, the incident acoustic field is mapped by using an interrogation laser beam to optically address different spatial points on the FP sensor. Two distinct implementations of this sensing concept have been described previously. The first and most common scheme is one in which a free-space focused interrogation laser is scanned across the surface of the FP sensor using a two axis galvanometer based conjugate scanner; this approach forms the basis of a range of non invasive pre-clinical [10–12] and clinical photoacoustic imaging [13] instruments. The second embodiment involves reading-out the FP sensor by delivering the interrogation beam via the individual cores of a rigid fibre bundle to realise a miniature endoscopic probe [14]. Both configurations have their limitations, particularly for clinical use. With the free-space FP scanner, the large size and weight of the galvanometers and conjugation optics makes it challenging to realise a compact lightweight hand-held imaging head. With the rigid fibre bundle approach, the range of endoscopic applications is limited to those where line-of-sight access is available. The limitations of both methods could be mitigated if the FP sensor is interrogated using a flexible fibre bundle. This would obviate the need for a bulky proximal end scanner enabling a compact lightweight probe head for non-invasive imaging to be realised. For endoscopic use, it would provide the flexibility required to reach anatomical sites that are inaccessible with a rigid probe.

The aim of the current study is to advance towards the above implementations by investigating the feasibility of interrogating the FP sensor using an inexpensive, commercially available flexible fibre bundle. This involved addressing several distinct technical challenges that do not arise with a free-space interrogated FP sensor. When using a fibre bundle, an optical scanner with higher spatial positioning accuracy is needed to individually address each core of the bundle. An additional optical system is also required in the form of a distal-end relay to achieve a sufficiently large FOV and an optimal interrogation beam spot size. Moreover, there are several potential sources of SNR degradation that are unique to a fibre bundle implementation, particularly when using commercially available fibre bundles [16] which are not designed for the commonly used 1500 nm − 1600 nm FP sensor interrogation wavelength range. In order to explore these issues and assess their impact on photoacoustic imaging performance, a bench-top experimental arrangement comprising an FP sensor interrogated using a flexible fibre bundle has been developed. This was used to assess optical and acoustic performance and compare it to that of a conventional free-space FP scanner [9]. In addition, the ability to acquire photoacoustic images using the system was demonstrated by imaging a range of tissue mimicking phantoms and *ex vivo* tissues.

## 2. Experimental setup

**Fig. 1.**
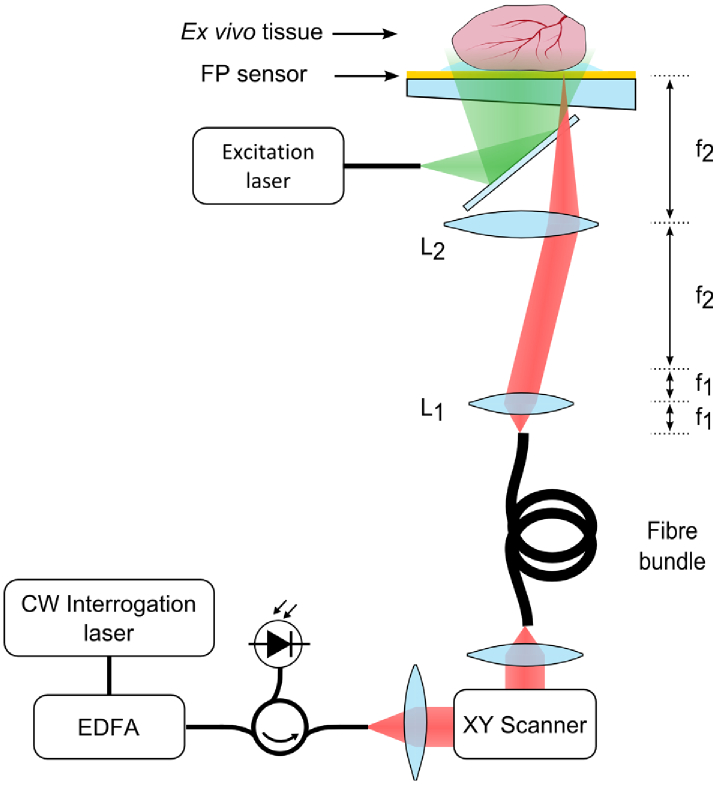
Experimental set-up illustrating the interrogation of the FP sensor using a flexible fibre bundle and telecentric lens relay system comprising $L_{1}$ and $L_{2}$.

In order to investigate the use of a flexible fibre bundle to read-out the FP sensor and establish its photoacoustic imaging performance, the experimental set-up in Fig. 1 was used. It comprises a flexible coherent fibre-optic bundle with an x-y galvanometer based optical scanner at its proximal end and a telecentric lens relay and a planar Fabry-Perot (FP) ultrasound sensor at its distal end. The scanner is used to scan the interrogation beam from core to core at the proximal end of the bundle in order to address different spatial points on the sensor and map the distribution of photoacoustic signals generated in the target. Previously we described a probe in which the FP sensor was deposited directly on to the tip of a 3.2 mm diameter rigid fibre bundle and a similar core-to-core scanning read-out approach employed [14]. However, depositing the FP sensor on to the tip of the 1.35 mm diameter flexible fibre bundle used in the current study would result in a small acoustic aperture that compromises the PA image quality. In addition, the small core diameter of the bundle used would produce an interrogation beam spot size that is smaller than desirable and compromise sensitivity. The use of the distal end telecentric relay between the fibre bundle tip and the FP sensor addresses these issues.

The fibre bundle (Schott Inc.) was a commercially available leached fibre bundle comprising 18,000 step-index fibre-optic cores (0.393 NA). The core/cladding diameter of each fibre was 6.7 µm/10.6 µm respectively. In addition a 0.17 µm thick $2^{nd}$ cladding remaining from the leaching process separated each fibre; thus the total core-to-core spacing was 10.6 µm. The diameter of the bundle was 1.35 mm and it was protected by a flexible plastic coating with a diameter of 2.2 mm. Since a laser source with narrow linewidth is used to interrogate the FP sensor, Fresnel reflections from the bundle endfaces interfere with the reflections from FP sensor and produce parasitic interference which acts as a source of noise. To suppress the detection of Fresnel reflections, both bundle endfaces were angle polished and wedged as described in Appendix A.

The telecentric lens relay comprises two achromatic doublet pairs and projects a magnified image of the bundle end face onto the FP sensor. Thus, when the interrogation beam is scanned over the proximal end of the bundle, corresponding points on the FP sensor are optically addressed. The relay serves two purposes. Firstly, it magnifies the lateral field-of-view (FOV) by a factor of $f_{2}/f_{1}$, where $f_{1}$ and $f_{2}$ are the focal lengths of lenses $L_{1}$ and $L_{2}$ as shown in Fig. 1; in this way a FOV arbitrarily larger than the bundle diameter can be realized by appropriate choice of the relay lens pair focal lengths. Secondly, it reduces the numerical aperture of the laser interrogation beam at the FP sensor by the same factor and reduces the beam walk-off, an important requirement for achieving high fringe visibility and finesse which are required for optimum sensitivity [15]. Experiments were conducted using magnifications 4.5× and 7.5×, attained by using focal lengths: $f_{1}$ = 16 mm (12.5 mm dia) and $f_{2}$ = 75 and 125 mm (50.8 mm dia). These magnifications resulted in 30 µm and 50 µm interrogation beam spot diameters at the FP sensor surface, and 6 mm and 10 mm diameter FOVs, respectively.

The interrogation laser was a CW wavelength-tunable external cavity laser (1550 nm center wavelength, Tunics T100s-HP, Yenista Optics) in conjunction with a 100 mW EDFA (Pritel FA-23). The high power provided by the EDFA is required to compensate for the losses incurred by the optical setup; the total one-way loss is approximately 90% and is made up of the Fresnel reflections at the fibre bundle endfaces and the relay optics combined (15%) and the intrinsic attenuation of the fibre bundle (85%). The latter is high because the core and cladding diameters of the specific bundle used are optimized for use at visible wavelengths [16]. However, at the interrogation laser wavelength of 1550 nm, the 6.7 µm core diameter and thin 1.78 µm cladding thickness results in weak confinement and thus significant attenuation. The output of the EDFA was coupled into the fibre cores via the x-y scanner and an achromatic doublet scan lens. The light reflected back from the FP sensor is coupled back into the bundle and directed via a single-mode fibre-optic circulator on to an InGaAs photodiode-amplifier unit with a bandwidth (−3 dB) extending from 0.5 MHz to 50 MHz connected to a 250 MS s^−1^ digitizer with a 125 MHz analogue bandwidth (not shown).

The FP sensor was fabricated on a 10 mm thick PMMA substrate by depositing a 15 µm thick transparent polymer spacer layer (Parylene C) between two dielectric mirror coatings. The mirror coatings were designed for high reflectivity in the spectral range 1500 nm to 1600 nm where FP sensor is interrogated, and high transmission in the visible and near-infrared region [9]. This allows for transmission of excitation wavelengths through the FP sensor for backward-mode photoacoustic imaging. The −3 dB acoustic detection bandwidth of the sensor used was 53 MHz [17].

Two different excitation laser sources were used to generate photoacoustic waves in the sample. For phantom experiments, the excitation source was a Q-switched Nd:YAG (Ultra, Big Sky) laser that emits 7 ns pulses at 1064 nm with 20 Hz pulse repetition frequency (PRF). To acquire images in biological tissues, a tunable (410 – 2100 nm) optical parametric oscillator (OPO) based laser system (Innolas Spitlight 600) emitting 7 ns pulses at 30 Hz PRF was used. In both cases, the excitation light was delivered via a multimode optical fibre. The output of the fibre was directed using a dichroic mirror through the sensor and on to the sample with a spot diameter of approximately 20 mm.

To acquire an image, the proximal end of the bundle was optically scanned from core to core. At each core position, the interferometer transfer function [9] (ITF), the relationship between optical power reflected from the FP sensor and interrogation wavelength, is acquired. The interrogation laser wavelength is then set to the point of maximum slope on ITF. Under these conditions, an acoustic wave (generated by the absorption of a pulse of laser light in the target) incident on the FP sensor will modulate its optical thickness. This results in a corresponding modulation in the reflected power of the interrogation laser beam which is transmitted back through the relay, along the fibre core and detected by the photodiode. Following acquisition of the photoacoustic signals from all 18,000 cores (which are arranged in a hexagonal pattern), the measured data is interpolated on to a uniform rectilinear grid before reconstructing the PA images using a time reversal algorithm [18,19].

For comparison purposes, a free-space FP scanner, similar to that described in [9] by Zhang et al., was used as a benchmark. With this system, the fibre bundle and the relay are not used and the sensor is directly read out by scanning a focused 50 µm diameter interrogation laser beam propagating in free-space over the surface of the FP sensor [9]; from here on, this will be referred to as the "free-space FP scanner". When using this scanner for comparison with the fibre bundle system, the interrogation laser power was adjusted so that the mean power recorded by the photodiode at the bias wavelength is approximately the same as that for the fibre bundle system; this compensates for the optical losses of the latter which are not present with the free-space scanner.

## 3. Results

In this section, the influence of the fibre bundle on the optical and acoustic performance of the FP sensor is investigated and compared to that achieved using the free-space FP scanner [9]. In addition, the photoacoustic imaging performance is assessed by measuring the line-spread function and imaging a range of tissue-mimicking phantoms and *ex vivo* biological tissues.

### 3.1 Comparison of fibre bundle and free-space interrogated FP sensor performance

#### 3.1.1 Round-trip coupling efficiency distribution

**Fig. 2.**
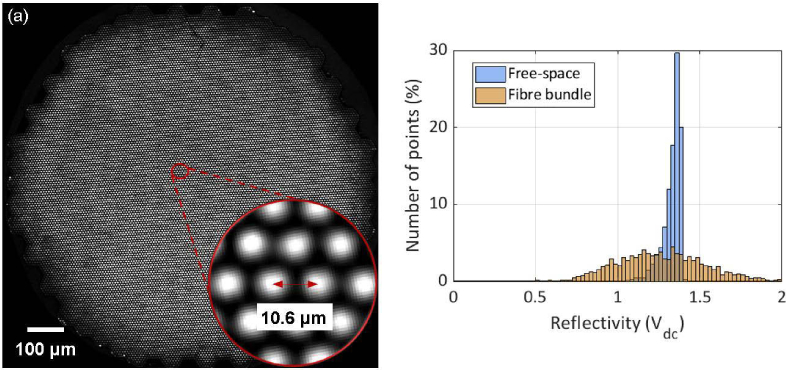
Relative round-trip coupling efficiency. (a) Scanned image of the fibre bundle using the set-up shown in Fig. 1. The greyscale intensity represents the measured photodiode voltage, $V_{dc}$, and provides a measure of the relative round-trip coupling efficiency of the system. (b) Histogram of data in (a) ($V_{dc}$ corresponds to the core centres) and that acquired from an identical scan of the same FP sensor over the same area and number of spatial points (18,000) using the free space FP scanner. The vertical axis in (b) represents the number of spatial points of value $V_{dc}$ expressed as a percentage of the total number of points scanned (18,000).

When using a fibre bundle to interrogate the sensor, it is desirable that the efficiency with which the interrogation beam is coupled into a specific core of the bundle, transmitted to the FP sensor via the relay and then re-coupled back into the core is similar for all cores. This requires a precision x-y scanner that can provide sub-micron positional accuracy over the entire 1.35 mm diameter of the bundle. It also requires that the distal end relay introduces negligible off-axis distortion to the beam. To assess the extent to which these requirements are met, the relative round-trip coupling efficiency of each core was measured. To achieve this, the interrogation laser wavelength was tuned to a point of zero derivative on the ITF. At this wavelength, the reflectivity of the FP sensor is independent of its optical thickness so the sensor acts as a mirror of spatially uniform reflectance. All 18,000 cores of the bundle were then individually addressed by scanning the interrogation beam over the proximal end of the bundle and recording the mean photodiode voltage ($V_{dc}$) at each core location. Figure 2(a) shows the results of such a scan where the greyscale intensity represents $V_{dc}$ and Fig. 2(b) shows a histogram of the same data. For comparison, this histogram also shows data acquired using the free-space FP scanner to scan the same sensor over the same area and number of spatial points. Figure 2(b) shows that for the fibre bundle case there is significant variation in $V_{dc}$ with the standard deviation 4 times higher than the free-space case. This is due to differences in core-to-core input coupling efficiency and off-axis aberrations in the lens relay which do not arise in the free-space case.

#### 3.1.2 ITF measurement

**Fig. 3.**
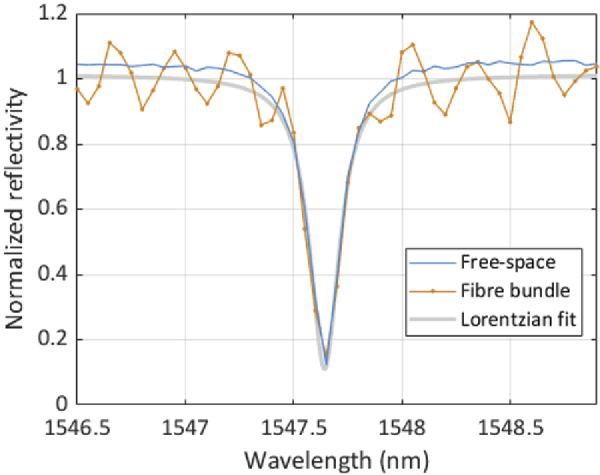
Interferometer transfer function (ITF) of the FP sensor interrogated by a single core of the fibre bundle (orange) and its Lorentzian fit (gray). The ITF of the same sensor interrogated by a free-space beam is also shown (blue) for comparison.

For high sensitivity, the design of the FP sensor (i.e., mirror reflectivities, spacer thickness, etc.) should be such that it provides high finesse and visibility. However, even if the FP sensor possesses these inherent characteristics, sensitivity can still be compromised if the measurement of the ITF is corrupted by noise introduced by the optical system. This is because such noise can distort the ITF to the extent that the optimum bias wavelength, $\lambda_{b}$ can no longer be accurately identified. This has not been observed to be a significant issue when using a free-space beam to interrogate the sensor [9]. However, use of a fibre bundle can introduce noise on the ITF that does not arise in the free-space case, especially if the core/cladding parameters are designed for visible wavelengths but longer wavelengths in the near infrared are used as in the current study; the fibre bundle that was used is specified for the 400 nm − 600 nm range but is used to transmit the 1500 nm − 1600 nm sensor interrogation light. At 1550 nm for example, the V number of the bundle is 5.34, and the number of guided modes is approximately 14; thus the fibre is weakly multimodal. Wavelength dependent interference between these multiple propagating modes can arise and introduce spurious baseline fluctuations in the ITF. Similar fluctuations can also be caused by wavelength-dependent optical coupling between adjacent cores. This is a distinct possibility given the small (1.78 µm) cladding thickness separating individual cores relative to the 1500 nm − 1600 nm wavelengths used. To investigate the impact of these influences, ITFs were acquired at each of the 18,000 core locations. For comparison, ITFs were also acquired at 18,000 points over the same area on the same sensor using the free-space FP scanner. Example ITFs acquired at a single point using both systems are shown in Fig. 3 and are of similar overall shape suggesting fibre bundle does not significantly distort the intrinsic ITF. This is further evidenced by the measured visibility and finesse obtained from the 18,000 ITFs acquired using both configurations. For the bundle, the mean visibility and finesse were 0.63 and 156.8, respectively; for the free-space scanner, they were 0.65 and 155.3. However, although these results suggest the fibre bundle does not negatively impact on the intrinsic interferometric characteristics of the sensor, Fig. 3 shows that it does introduce significant baseline fluctuations, most likely due to the above mentioned inter-modal interference and crosstalk effects. In principle, this could compromise the accurate identification of $\lambda_{b}$. However, previous experience using a rigid bundle [14], which is afflicted by a similar level of ITF baseline noise, suggests this is not significant if a noise-free ITF, obtained by fitting a Lorentzian to the measured ITF, is used to determine $\lambda_{b}$ - as described in section 2, this is the approach employed in the current study.

#### 3.1.3 Acoustic SNR

For a given FP sensor interrogated using a fibre bundle, the critical question is whether the acoustic SNR will be lower than that achieved when using a free-space beam to interrogate the same sensor. To assess this, the output of a 25 mm diameter, 3.5 MHz planar ultrasound transducer that emitted a plane wave with an amplitude variation of less than 5% over a 15x15 mm^2^ area was directed on to the FP sensor. The peak positive amplitude of the acoustic waveform (which is taken to represent the signal) and the RMS noise (over a 20 MHz measurement bandwidth) were measured at different points on the sensor using firstly the fibre bundle and then the free-space FP scanner [9]. In both cases, an identical circular area of 10 mm diameter was scanned and measurements were made over the same number of spatial points (18,000). For the free-space FP sensor scan, the interrogation laser power was adjusted so that the mean dc voltage measured by the photodiode at the bias wavelength was approximately the same as the fibre bundle case to ensure a fair comparison.

**Fig. 4.**
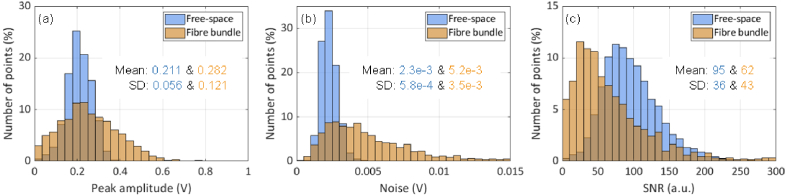
Histograms of (a) signal, (b) noise and (c) signal-to-noise ratio (SNR) for fibre bundle and free-space interrogated FP sensor configurations. In both cases the FP sensor was interrogated at 18,000 different points over a circular area of 10 mm diameter. The vertical axes represents the number of spatial points expressed as a percentage of the total number of points scanned (18,000).

The signal, noise and SNR distributions are shown in the histograms in Fig. 4. First consider the signal distribution (Fig. 4(a)). The mean signal is similar for both free-space and fibre bundle configurations. However, for the fibre bundle, the variation is larger due to the variations in round-trip coupling efficiency which do not apply to the free-space case as described in section 3.1.1 (Fig. 2(b)). The noise distribution is shown in Fig. 4(b). This shows that the mean noise is higher for the fibre bundle system than the free-space case. It was observed that this noise is broadband and encompasses the ultrasonic frequency range and thus cannot be filtered out. Its high frequency nature suggests it is unlikely to originate from low-frequency vibrations. The most likely source is parasitic interference between two or more optical fields with a pathlength difference on a scale comparable to the laser coherence length. Under these conditions, phase noise arising from the finite laser linewidth is converted to intensity noise. There are several possible sources of such noise. It could arise from superposition of the light reflected from the front and distal ends of the fibre which involves a significant pathlength difference (>1 m). However, the wedge on the proximal fibre bundle end-face ensures that the magnitude of the front-end reflection that is coupled in to the circulator and detected by the photodiode is negligible. Parasitic interference arising from these fibre-endface reflections is therefore unlikely to be significant. Although the wedge on the distal end of the bundle also suppresses the Fresnel reflection, the high NA of the fibre and the relatively small wedge angle (8.3° may result in a non negligible reflection. This reflection and that from the FP sensor could then form a parasitic interferometer with a multi-cm pathlength difference that introduces noise. Other possible noise sources are inter-modal interference and coupling between adjacent cores, although in both cases the scale of the pathlength differences involved is likely to be small compared to the laser coherence length. The SNR distributions in Fig. 4(c) reflect both signal and noise distributions. The fibre bundle SNR is lower with a mean of 62 compared to 95 for the free-space case. Since the mean signal is similar for both cases (Fig. 4(a)), the reduced SNR is largely a consequence of the higher noise introduced by the bundle.

Finally, given the propensity for inter-modal interference and cross coupling effects, both of which are sensitive to environmental influences, the fibre bundle was repeatedly twisted, bent and shaken while monitoring the acoustic signal in real time on an oscilloscope. No significant changes in the acoustic signal amplitude were observed.

### 3.2 Photoacoustic imaging performance

In this section, the ability of the system to acquire photoacoustic images is assessed by scanning tissue mimicking phantoms and vascularized *ex vivo* tissues.

#### 3.2.1 Spatial resolution

**Fig. 5.**
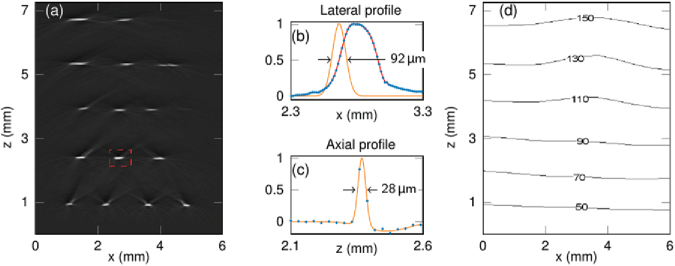
Spatial resolution of the fibre bundle FP sensor imaging system. (a) Reconstructed PA image showing ribbon cross-sections at different depths. (b) Lateral and (c) axial profiles through the ribbon feature identified by the dotted red rectangle in (a), respectively. (d) A contour plot showing the lateral spatial resolution in the x-z plane.

The lateral and vertical spatial resolution was evaluated by imaging five rows of absorbing ribbons distributed over a depth of 7 mm that provide a step edge in the lateral direction. Deionized water was used to provide acoustic coupling between the FP sensor and the ribbon phantom. An excitation wavelength of 1064 nm and a fluence of 20 mJ cm^−2^ was used. The interrogation beam was scanned in 2D over the proximal face of the bundle in order to map the spatial distribution of the PA waves incident on the FP sensor at the distal end; the 4.5× lens relay was used for this experiment. A 3D PA image was reconstructed from this data. Figure 5(a) shows an x-z slice from the reconstructed 3D PA image where ribbon cross-sections are clearly visualized up to 7 mm in depth. The lateral resolution was determined by taking the FWHM width of a Gaussian fit to the derivative of lateral profile (Fig. 5(b)). The lateral spatial resolution at a depth of 1 mm was 57 µm and gradually decreased to 150 µm at 7 mm in depth, as shown in the contour plot (Fig. 5(d)), a consequent of the limited view provided by the scan area. The axial line spread function was determined by taking the FWHM width of a Gaussian fit to the axial profile (Fig. 5(c)). It was found to be 28 µm and largely spatially invariant over the x-z plane. The measured axial and lateral resolutions are broadly consistent with those previously obtained using free-space FP based scanners [9–11]. This suggests that image degradation due to crosstalk between adjacent cores and any distortions in the mapping of the hexagonal pattern of the fibre cores on to the FP sensor plane by the relay optics is not significant.

#### 3.2.2 Arbitrary shaped phantoms

**Fig. 6.**
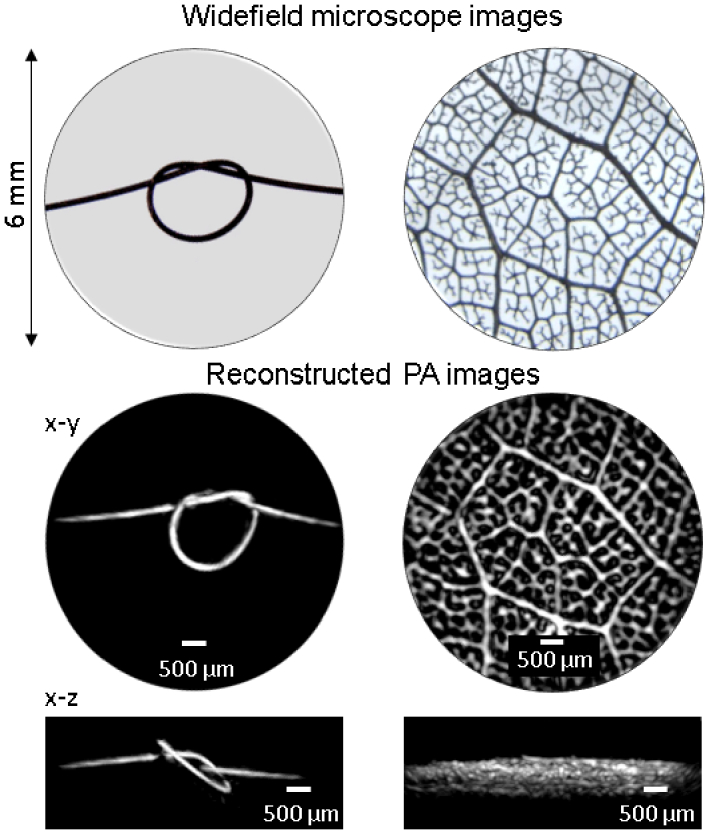
PA images (6 mm aperture) of arbitrary shaped phantoms. Top panel: widefield microscope images of a synthetic hair knot and leaf skeleton phantom coated in India ink. Middle and lower panels: reconstructed PA images of the phantoms shown as maximum intensity projected along the x-y and x-z planes.

The three-dimensional imaging capability of the system was demonstrated by imaging two arbitrary shaped phantoms. The top panel in Fig. 6 shows the widefield microscope images of a synthetic hair knot, and a leaf skeleton phantom coated in India ink. These phantoms were immersed in a deionized water bath about 1 mm away from the FP sensor surface. PA signals were acquired using a 1064 nm excitation wavelength and a fluence of 20 mJ cm^−2^. The reconstructed PA images of the phantoms, maximum intensity projected along the x-y and x-z plane, are shown in the bottom panel of Fig. 6. The structural features of synthetic hair knot and twisted ribbons phantoms are accurately reproduced in the PA images. The intricate veins of the leaf skeleton phantom are also clearly visualized in the PA image.

#### 3.2.3 *Ex vivo* tissues

To demonstrate the high-resolution imaging capability in biological media, *ex vivo* tissues with vascular architectures of different spatial scales were scanned using the system with the 7.5× relay to provide a 10 mm circular FOV.

**Fig. 7.**
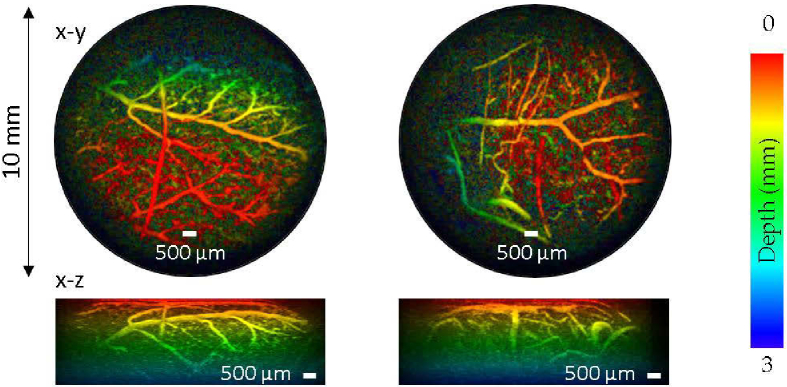
PA images (10 mm aperture) of an *ex vivo* duck chorioallantoic membrane (CAM) where microvasculature is clearly visualized. The images are-coded according to the depth and maximum intensity projected along the x-y and x-z planes. Laser excitation wavelength: 590 nm, fluence: 18 mJ cm^−2^.

First, the chorioallantoic membranes (CAM) from fourteen day-old duck eggs were imaged using 590 nm excitation wavelength and 18 mJ cm^−2^ fluence. The procedure was not considered to require Home Office Regulation (Animals (Scientific Procedures) Act 1986) as the duck embryos had not reached the last third of gestation (normal gestation period is 28 days) and the embryo was killed before the start of the final third of the incubation period. Figure 7 shows the maximum intensity projected PA images of the duck CAM, colour-coded according to the depth. The images show the dense blood vasculature of the CAM, which is the outermost extra-embryonic membrane that is highly vascularized for gaseous exchange and calcium transportation between the embryo and its environment. The fine network of blood vessels in the CAM, some as small as 50 µm across, are clearly visualized in the PA images.

**Fig. 8.**
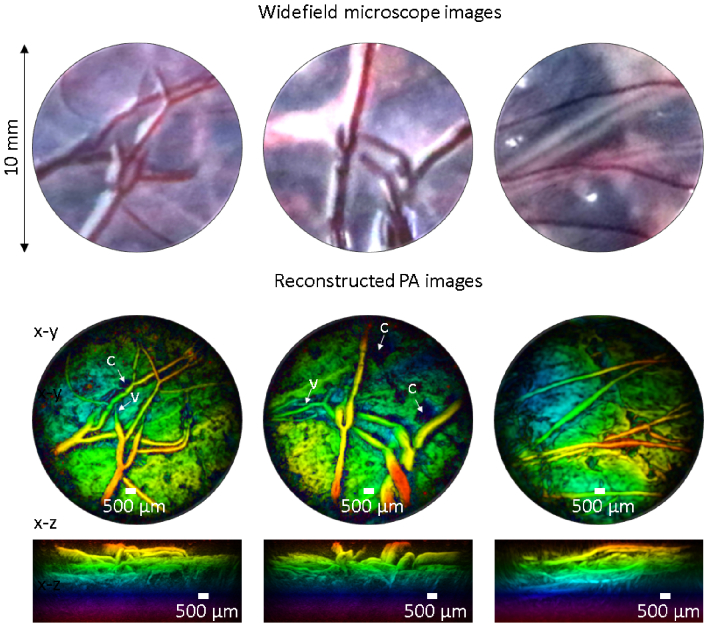
PA images (10 mm aperture) acquired at three locations on an *ex vivo* term normal human placenta. Top panel: widefield microscope images from the fetal side of the placenta where chorionic (fetal) vessels are visualised. Middle and lower panels: 3D PA images of the same area as top panel,-coded according to the depth and maximum intensity projected along the x-y and x-z planes. Locations marked by letter v and c indicate areas where sub-surface chorionic vessels and calcium deposits are visualized, respectively. Laser excitation wavelength: 590 nm, fluence : 18 mJ cm^−2^.

An *ex vivo* normal term human placenta with larger vessels was also imaged. The placenta was collected with written informed consent after a caesarean section delivery from a healthy term pregnant woman at University College London Hospital (UCLH). The Joint UCL/UCLH Committees on the Ethics of Human Research approved the study (14/LO/0863). At delivery, the umbilical cord of the placenta was clamped to preserve blood in the fetal chorionic microvasculature. After delivery the amniotic membrane was stripped from the chorionic surface. A water-based gel was used for acoustic coupling, and PA images were acquired using a 590 nm excitation wavelength and 18 mJ cm^−2^ fluence. The top panel in Fig. 8 shows 10 mm diameter widefield microscope images from the chorionic surface of the placenta, and the bottom panel shows the 3D PA images from the same area. Locations marked by letter v indicate areas where sub-surface chorionic vessels are visible in the PA images but are not visible in the widefield microscope images of the chorionic surface. The tissue surrounding the chorionic vessels has strong PA contrast, which originates from the dense fetal villous capillary architecture. The PA images also show patches in the surrounding tissue where there is a pronounced absence of PA contrast. These areas of anomalous negative contrast, marked with letter c, are likely to be the sites of calcium deposits [20] or areas of infarction in the chorionic plate arising from chronic vascular placental impairment; the latter are commonly seen in term placentas, even those with a normal outcome and are classified according to a "Grannum grade" which describes their depth and distribution through the placental tissue [21].

## 4. Discussion

This study has explored the use of a flexible coherent optical fibre bundle to interrogate a FP ultrasound sensor. When using the fibre bundle, it was found that variations in core-to-core round trip coupling efficiency result in acoustic sensitivity variations over the sensor scan area that are significantly greater than observed when using a free-space interrogation beam (Fig. 4(a)). In principle, this can introduce spatial variations in image SNR but in practice this was not observed to any significant extent. This is because in widefield photoacoustic tomography mode, each reconstructed image pixel is the superposition of a significant proportion of the total number of photoacoustic signals detected over the entire FOV. This has a smoothing effect such that the spatial variation of the detection sensitivity is not mapped directly on to the reconstructed image but averaged out. Nevertheless, if required, there is scope for improvement since these variations in sensitivity most likely arise from misalignments between the focused interrogation beam and the cores at the proximal end of the bundle or off-axis aberrations in the relay optics. They could be reduced by optimising the design of the scanner optics and control hardware to improve positioning accuracy and refining the relay lens design to reduce aberrations. The fibre bundle system also exhibits high optical attenuation. As demonstrated, this can be inexpensively compensated for by using an EDFA to increase the interrogation laser power. If required however, use of an EDFA could be avoided by using a bundle designed for low loss propagation at telecom wavelengths and depositing anti-reflection (A/R) coatings on the fibre endfaces and the relay lenses to reduce Fresnel reflection losses.

The fibre bundle also introduces noise that is not present with the free-space FP scanner. As described in section 3.1.2, it introduces baseline fluctuations on the ITF. However, this appears not to be a limiting a factor since it does not compromise accurate identification of the optimum bias wavelength. More problematic however, is the increased broadband noise that the fibre bundle introduces since it encompasses the ultrasonic frequency range and thus reduces acoustic SNR; as Fig. 4(c) shows, the mean SNR is approximately a factor of 1.5 lower than obtained with a free-space FP scanner. Further investigation is required to establish the precise origins of this increased noise. If it is primarily due to parasitic interference between the reflections from the fibre-bundle end-face and the FP sensor, then increasing the angle of the distal-end wedge and depositing an anti-reflection coating on to the tip of bundle would reduce it. Noise due to inter-modal interference could be minimised by reducing the number of propagating modes (preferably to one) by designing the input coupling optics to provide a lower NA and increasing the scanner positional accuracy so that the focused interrogation beam can be aligned more precisely with the axes of the cores. If the noise is due to core-to-core cross coupling, then increasing the cladding thickness, albeit at the cost of a non-trivial redesign of the bundle, could reduce it. Indeed, most of the above limitations are a consequence of using a commercially available fibre bundle designed for use at visible wavelengths. They could be mitigated by using a fibre bundle designed to provide low loss single mode operation at 1550 nm.

The photoacoustic imaging performance was also evaluated. Image resolution and spatial fidelity could be compromised by spatial distortion introduced by aberrations in the distal-end lens relay system or cross-coupling between adjacent cores. However, no evidence of this was observed from the phantom studies undertaken. The lateral spatial resolution was 50 µm at a depth of 1 mm from the sensor surface and gradually decreases to 150 µm at 7 mm in depth. The axial resolution is 28 µm, which is largely invariant across the entire field of view. Both estimates are consistent with those obtained previously with free-space FP scanners [9]. This is further evidenced by the high quality of PA images of the vasculature in *ex vivo* duck embryo and human placenta where 3D vascular structures are clearly differentiated from the surrounding tissue.

## 5. Conclusions

In summary, this study has shown that, when using a standard commercially available flexible fibre bundle to spatially the map the output of the FP sensor, it is possible to acquire high quality PA images, comparable in terms of resolution and spatial fidelity to those obtained with a free-space FP scanner. The acoustic SNR when using a fibre bundle is lower due to higher noise, most likely due to the distal end fibre-end reflection and the non-ideal propagation characteristics of the bundle. Nevertheless, even with these non-optimal characteristics, the SNR was sufficient to acquire photoacoustic images of tissue with a penetration depth sufficient for superficial non invasive and endoscopic imaging applications. Moreover, depending on the noise source, there is scope for improvement by modification of the distal end of the bundle-end-face, improved design of the scanner optics and using a bundle designed for single mode operation in the 1500 nm to 1600 nm wavelength range. Successful use of a flexible fibre bundle to interrogate the FP sensor would considerably broaden its applicability to biomedical photoacoustic imaging and ultrasound field mapping. Most obviously it would pave the way to miniaturized, flexible photoacoustic imaging probes for endoscopic use, hand held non-invasive clinical scanners for imaging the skin or applications where an electrically passive, non-ferrous imaging head is required, for example for photoacoustic or ultrasound imaging in an MR scanner.

## Figures and Tables

**Fig. 9.**
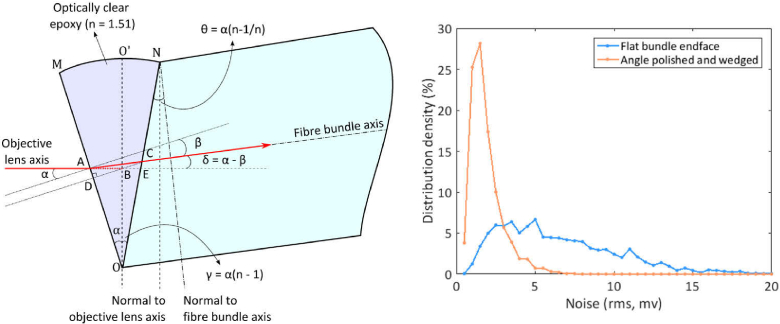
(left) Ray diagram illustrating the relationship between angle of the wedge and angles at which the fibre bundle endface is polished and tilted to suppress the detection of Fresnel reflections. (right) Histogram plot shows RMS noise floor of the FP sensing elements before (blue) and after (orange) angle polishing and wedging of the bundle endfaces. The noise was measured by scanning 3,000 cores in the centre of the bundle with 20 MHz detection bandwidth.

## Appendix A: minimizing the detection of Fresnel reflections from bundle endfaces

To suppress the Fresnel reflections, both bundle endfaces were polished at an angle $\theta \;>\;\sin^{-1}(NA)$, where NA is the numerical aperture of the objective lens that focuses the interrogation beam. However, this poses the challenge that only a small part of the 1.35 mm diameter bundle endface remains in the focal plane of the objective lens [22]. This was resolved by applying an optically clear epoxy to the proximal endface and polishing at an angle $\alpha =\frac{\theta }{\text{n}-1/\text{n}}$ (where n is the refractive index of the epoxy) to form a counter wedge, which shifts the focal plane to the angled endface. In addition, at the proximal end, the bundle needs to be tilted by an angle δ, so that the axis of the incident focused beam is aligned with the axis of the fibre cores to achieve maximum coupling efficiency. The relation between angle of the wedge (α) and the angles at which the bundle endface was polished (θ) and tilted (δ) are derived using the ray diagram in Fig. 9. According to the law of refraction, sin⁡α=nsin⁡β which is equivalent to AD/AB=nAD/AC. Assuming the paraxial approximation, AC≅AE≅AB+BE, which gives the relation BE≅AB(n−1), where BE is the apparent shift of the focal plane due to the epoxy wedge from OO’ (which represents the location of the focal plane in the absence of the wedge) to ON. This angular shift is given by:

(1) $$\tan\gamma =\frac{BE}{BO}≅\frac{AB}{BO}(\text{n}-1)≅\tan\alpha (\text{n}-1),\;or\;\gamma ≅\alpha (\text{n}-1)$$

The bundle endface needs to be positioned at an angle α(n−1) to remain in focus, and additionally tilted by an angle δ for maximum coupling efficiency as described above. These conditions lead to the angle, θ, at which the bundle endface needs to be polished:

(2) $$\theta =\delta +\gamma ≅\alpha -\frac{\alpha }{\text{n}}+\alpha (\text{n}-1)≅\alpha (\text{n}-1/\text{n})$$

The proximal end of the fibre bundle was scanned with a 0.12 NA objective lens, so the bundle endface and the epoxy (n=1.51) surface was polished at angles 7° and 8.3°, respectively, and the endface was tilted 2.8° from the objective lens axis. The histogram plot in Fig. 9 compares the RMS noise voltage recorded at the output of the photodiode-amplified unit when the FP sensor was interrogated using the fibre bundle prior to (blue) and after the endfaces were angle polished and wedged (orange). On average, the baseline noise reduced by a factor of 3.2 when the Fresnel reflections were suppressed thereby significantly improving the acoustic sensitivity.
